# Foreign bodies in the thyroid gland and foreign body reactions in the post-thyroidectomy bed

**DOI:** 10.3389/fendo.2026.1826008

**Published:** 2026-09-16

**Authors:** Przemysław Paul, Łukasz Obołończyk, Krzysztof Sworczak

**Affiliations:** 1 Department of Internal, Occupational, Metabolic and Endocrine Diseases, Medical University of Gdańsk, Gdynia, Poland; 2 Department of Endocrinology and Internal Diseases, Medical University of Gdańsk, Gdańsk, Poland

**Keywords:** foreign bodies, thyroid gland, foreign body migration, foreign body reaction, thyroidectomy, postoperative complications, thyroid surgery

## Abstract

Foreign bodies (FBs) in the thyroid gland are very rare and may create diagnostic and therapeutic problems. So far, there have been a few review articles concerning only ingested FBs in the thyroid gland, while other mechanisms remain poorly studied. FB reactions mimicking thyroid regrowth in the post-thyroidectomy bed may also pose a diagnostic challenge for clinicians due to their rarity. The article discusses ingested, traumatic, and iatrogenic FBs in the thyroid gland. Our search generated 56 relevant articles with 63 total cases of FBs in the thyroid gland. The review highlights the clinical presentation, the most useful diagnostic methods, and treatment modalities. The vast majority of the FBs in the thyroid gland were fish bones (63.5%). The analysis shows that over one-third of the patients may be misdiagnosed at the initial evaluation. Moreover, tissue responses to the foreign materials in the post-thyroidectomy bed mimicking actual FBs are described with particular attention to the diagnostic imaging and histopathologic findings. FBs in the thyroid gland and FB reactions in the post-thyroidectomy bed represent rare challenges that require prompt management. Although these medical conditions are very rare, they can lead to many diagnostic difficulties, which may influence the patient’s care.

## 1 Introduction

A foreign body (FB) can be defined as an object or piece of extraneous matter that has entered the body. FBs can be introduced into the organism through various routes, including intentional, accidental, or iatrogenic means (1). FBs as a medical problem have accompanied mankind since the dawn of time. Back in the 6th century BC, the ancient Greek fabulist Aesop wrote a fable about a crane that helped remove a bone wedged in a wolf’s esophagus (2). Since then, many examples of FBs in different parts of the human body have been reported. Notably, FBs in the neck can also be found in the art. Probably the most common example is an arrow, which appears in many portraits of Saint Sebastian and Saint Christina of Bolsena. Two examples of such are shown in **Figures 1** and **2**.

**Figure 1:**
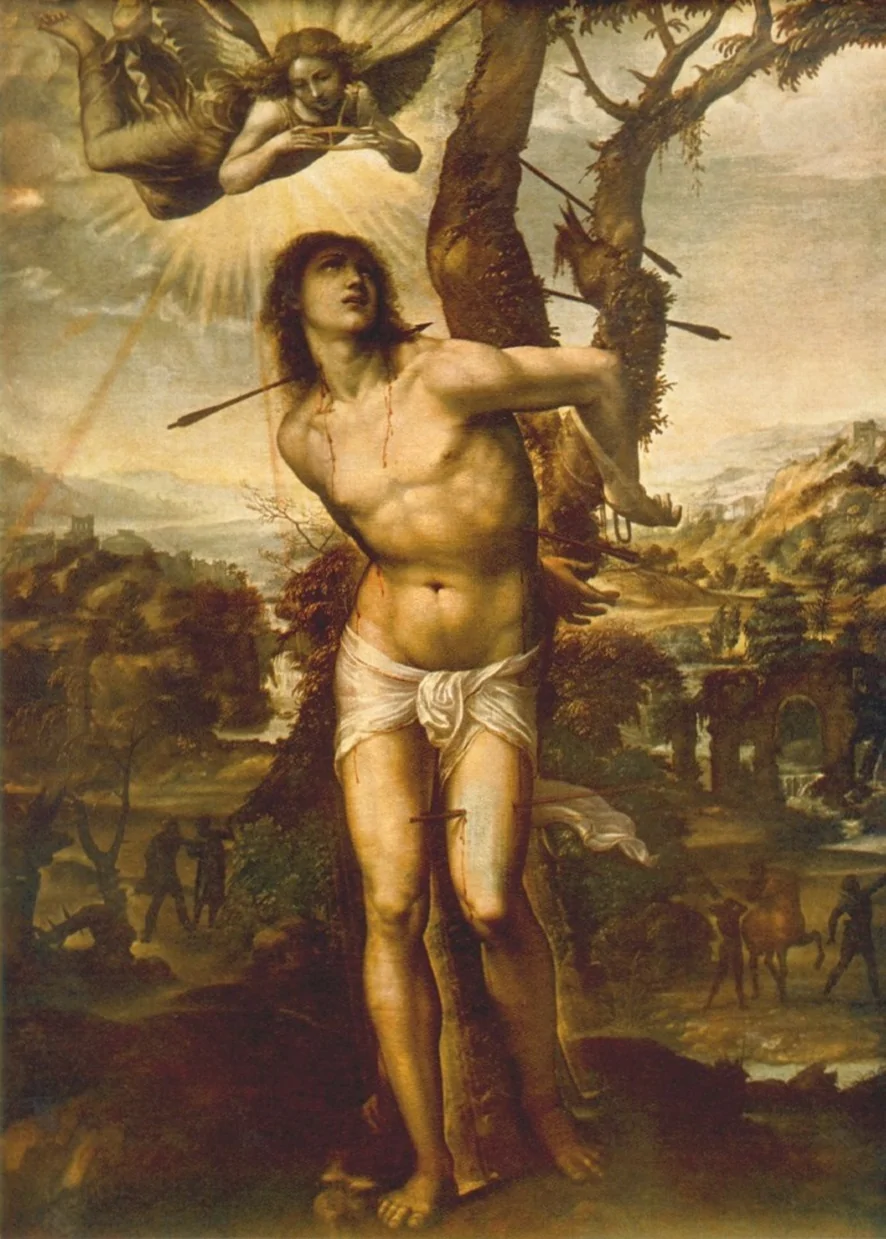
Martyrdom of Saint Sebastian, Giovanni Antonio Bazzi, known as Il Sodoma, 1525. Oil on canvas. Gallerie degli Uffizi, Florence, Italy. Public domain. Source: Wikimedia Commons.

**Figure 2:**
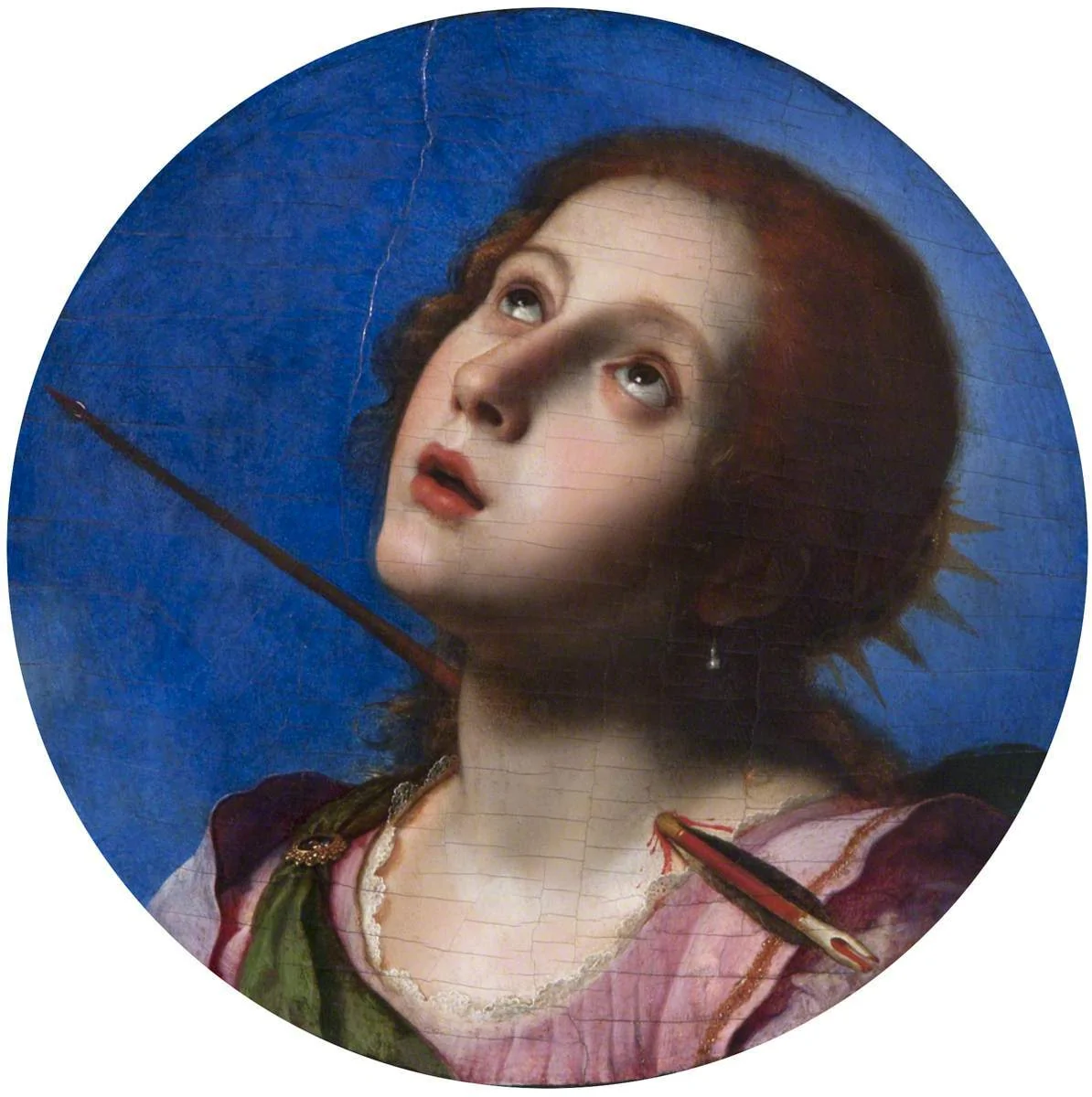
*Saint Christina of Bolsena*, by Carlo Dolci, mid-17th century. Oil on panel. Eastern Museum, Kedleston Hall, Derbyshire, England. Public domain image. Source: Wikimedia Commons.

The most comprehensive literature on FBs covers three organ systems: respiratory, digestive, and genitourinary tracts. In the adult population, accidentally ingested FBs are usually fish bones, bones of other animals, and dentures (3). The variety of FBs inserted into the genitourinary tract surpasses imagination and includes all types of objects (4). In contrast, there are human organs where finding an FB is uncommon. Examples include the Eustachian tube (5), the urachus (6), or the thyroid gland.

The main aim of this article is to inform readers about the types of possible FBs in the thyroid gland and FB reactions in the bed after thyroidectomy, the main risk factors, symptoms, useful imaging modalities that can help avoid misdiagnosis, treatment methods, and outcomes. So far, there have been few review articles concerning migratory FBs in the thyroid gland. This term refers to the situation in which the FB migrates through the esophageal wall (7). An ultrasound (US) examination of the neck usually shows the position of the esophagus and its relationship to both lobes of the thyroid gland (**Figure 3**).

**Figure 3:**
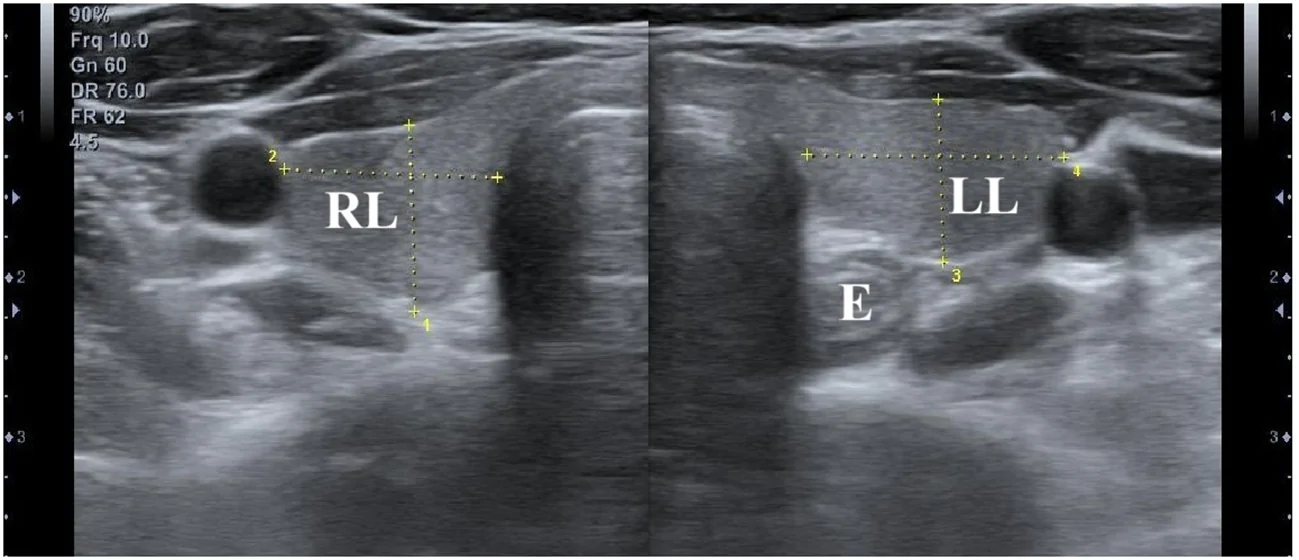
Ultrasound image of the thyroid gland. Transverse plane. RL, right lobe of the thyroid gland; LL, left lobe of the thyroid gland; E, esophagus.

To the best of our knowledge, our article is the first one summarizing all reported FBs in the thyroid gland and FB reactions in the bed after thyroidectomy described in the literature.

## 2 Materials and methods

In this paper, we review the reported cases of FBs in the thyroid gland and FB reactions in the post-thyroidectomy bed. The review is based on an electronic literature search performed in the PubMed, Scopus, Embase, and Web of Science databases through December 2025 with the following key terms: thyroid gland & FB; thyroid gland & FBs; thyroid gland & FB reaction; thyroidectomy & FB reaction. Publications in English reporting FBs in the thyroid gland and/or adjacent to the thyroid gland and FB reactions in the post-thyroidectomy bed were included. In addition, reference lists of the retrieved publications were reviewed to find additional relevant studies. Statistical analyses were performed using available data only. Cases with missing data or qualitative descriptions that could not be converted into numerical numbers were excluded from the analyses. This article is a review of previously published data and does not contain any new studies with human or animal participants. Accordingly, institutional review board approval was not required.

## 3 Results

Our search generated 56 relevant articles with 63 total patients. Detailed summaries of all reported cases of the FBs in the thyroid gland are provided in the **Supplementary Table 1**. The **Supplementary Table 1** contains data on the year and country of publication, type of FB, its size and location, the age and sex of the patients, main symptoms, time from first symptoms to presentation, information on whether the patient was initially misdiagnosed, complications, main treatment, and diagnostic methods.

The vast majority of FBs were fish bones, accounting for 63.5% of cases. The second largest group consisted of metal needles/wires (15.9%), and the third one was polytetrafluoroethylene (commonly known as Teflon) (6.3%). We have found three cases of chicken bones and also cases of a lamb bone, a tooth, a piece of glass, ultrasound gel, a minitracheostomy tube, and vegetable matter (**Table 1**).

**Table 1:** Types and quantities of previously reported cases of foreign bodies (FBs) in the thyroid gland (1, 7–61).

Type of FB	Quantity of cases	Percentage of cases [%]
Fish bone	40	63.5
Metal needles/wires	10	15.9
Polytetrafluoroethylene	4	6.3
Chicken bones	3	4.8
Lamb bone	1	1.6
Tooth	1	1.6
Piece of glass	1	1.6
Vegetable matter	1	1.6
Ultrasound gel	1	1.6
Minitracheostomy tube	1	1.6

Among 63 cases, 54 were female (85.7%). The mean age was 52 years, with the minimum being 12 and the maximum being 80. FB size was available for 45 of 63 cases. The mean length of an FB was 27.2 mm. The location of the FBs was available for 62 of 63 cases. The left thyroid lobe was affected in 31 cases (50.0%), the right lobe in 30 cases (48.4%), and the isthmus in one case (1.6%). Chief complaints were available for 58 of 63 cases. The most frequently reported symptoms were neck pain (50.0%), followed by neck swelling (32.8%) and odynophagia (25.9%). Dysphagia and FB sensation were less common and accounted for 24.1% and 17.2% of patients, respectively. Among 52 cases with available quantitative data, the median interval between symptom onset and hospital presentation was 9.5 days. This time varied from a few hours to a few years. Notably, misdiagnosis at the initial evaluation was reported in 19 of 55 cases with available data (34.5%). Information regarding complications was available for 60 of 63 cases. Over half of the patients did not experience any complications of the FB in the thyroid gland (53.3%). The most reported complication was thyroid abscess, which was described in 38.3% of cases. Two patients with thyroiditis, one with hyperthyroidism, one with hematoma and one with a skin fistula were also revealed. The most commonly used diagnostic method was computed tomography (CT) (89.3%), followed by different types of endoscopy (78%) and plain X-rays of the neck (51.8%). The US was applied in 35.7% of patients and barium fluoroscopy in 7.1%. Thyroid-preserving surgical removal of the FB was the most frequently used treatment method, accounting for 58.7% of cases. More than a quarter of the patients required hemithyroidectomy (27%). Minimally invasive endoscopic methods could be used only in four situations (6.3%). One case was treated only with an antibiotics course. In the single case of teflonoma, just observation was advised.

## 4 Discussion

### 4.1 Foreign bodies in the thyroid gland

Migratory FBs are the most commonly reported FBs affecting the thyroid gland. The ingestion of FBs results in gastrointestinal perforation in less than 1% of patients (62). The mechanisms that cause FBs to pierce the esophagus are unknown. It has been postulated that the combination of peristalsis, the contraction of the cricopharyngeal muscles during swallowing, and neck movement play dominant roles in this process (63). Due to the solid barriers of the anterior (trachea) and posterior (cervical vertebra) regions of the esophageal wall, an FB might penetrate only laterally, for example, towards the level of the right or left lobe of the thyroid gland (36). Foo details that the typical shape of a migratory FB is “spear-like/needle-like” (14). Other shapes like triangular or “T-shaped” are usually entrapped at the cricopharynx. Watanabe et al. reveal that fish bones usually become lodged in the palatine tonsil, the base of the tongue, the vallecula, the pyriform sinus, and the esophagus; however, its penetration is rare (34). Dentures are considered to be one of the major risk factors for accidental ingestion of an FB (64). Having dentures reduces the perception of sharp edges (e.g., fish bones or metal wires) in the palate and tongue. Other risk factors in adults include pre-existing esophageal pathology, older age, and neurological disease (65). Masuda et al., in their publication, point out that patients with goiter may be more vulnerable to migratory FB to the thyroid gland due to the increased contact area with the pharyngeal space and esophagus (25) (**Figure 4**).

**Figure 4:**
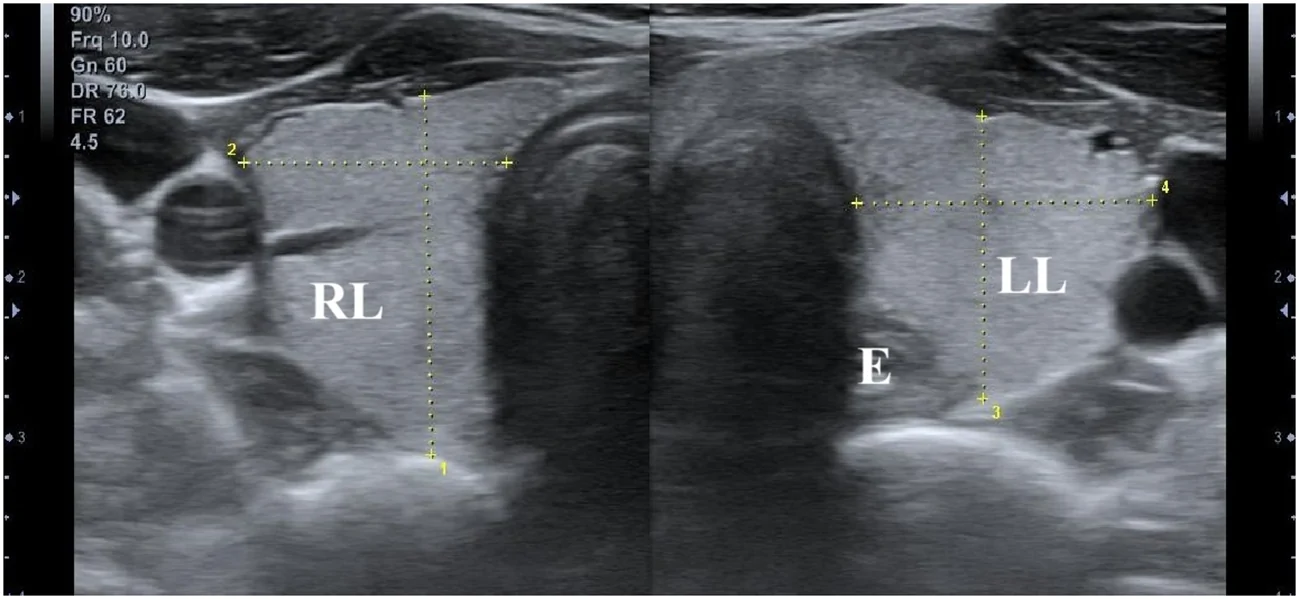
Ultrasound image of the goiter. Transverse plane. RL, right lobe of the thyroid gland; LL, left lobe of the thyroid gland; E, esophagus.

Foo, in his publication, highlights that cultural eating habits may also play a significant role as a risk factor (14). This includes the preference for bony fish instead of filleted fish, meals that contain broken bone chips, and the use of chopsticks, which worsen the feeling of fish bones. This point is consistent with the results of the review. Our statistics show that the average person with FB in the thyroid gland is over 50 years old. Additionally, the majority of accessible cases in the literature come from Asian countries (61.9%), where eating bony fish and using chopsticks is more common than on other continents. According to the data we have collected, there is a clear predominance of females in the incidence of FBs in the thyroid gland (85.7%), which is coherent with other review articles concerning migratory FBs in the thyroid gland (30, 32, 41). Hohman et al. pointed out that the cause of this predominance could be multifactorial, including mastication and deglutition habits, mucous membrane resilience, and differences in diet (30). Wu et al., in their publication, prove that migratory FBs more often penetrate to the left lobe of the thyroid gland because the esophagus physiologically is slightly deflected to the left side of the trachea (41) (see **Figure 3**). Unlike in the source mentioned above, our data show almost even distribution of FBs in both lobes of the thyroid gland. It should be noted that our analysis goes beyond migrating FBs. The duration of the migration of FBs in soft tissues is probably different in every case. However, Chen et al. reported that with the use of repeated CT, the authors tracked a metal wire that pierced the esophagus. It finally reached the right lobe of the thyroid gland 10 days after ingestion (36). In this place, it is worth emphasizing that the European Society of Gastrointestinal Endoscopy (ESGE) recommends urgent (preferably within 2h, at least within 6h) therapeutic esophagogastroscopy for swallowed FBs (66). The authors state that the risk of major complications (i.e., aortoesophageal fistula, retropharyngeal abscess, perforation of the mediastinum) increases over 14 times with FBs impacted over 24h in the esophagus.

The most frequently reported symptom of the FB in the thyroid gland is neck pain. According to Sun et al., the differential diagnosis of the persistent neck pain includes epiglottic cysts, tonsillitis, and acute epiglottitis (52). Noteworthy is that subacute thyroiditis might be unilateral as well as mimic FBs. What is alarming is that over one-third of the patients collected in our review were initially misdiagnosed and discharged home. After analyzing the material, the main reason for that seems to be negative results from first-line diagnostics. If the FB penetrates the esophagus, endoscopy might be negative. The lateral neck radiograph is often the initial imaging method due to its high availability and low price. Unfortunately, interpretation of the plain X-ray is not always simple, because FBs might be mistaken for calcified physiological structures (32). Moreover, fish bones, which are the most common FBs in the thyroid gland, may be radiolucent or radiopaque. In **Table 2**, some examples of fish species with radiolucent and radiopaque fish bones are presented.

**Table 2:** Radiopacity of different types of fish bones in plain X-rays (67, 68).

Degree of opacity	Radioopaque	Radiolucent
Types of fish	Hardtail scad Snapper Tuna Pangas Catfish Seabass Croaker Herring Grouper Bream Mullet Whiting Salmon Barramundi	Mackerel Short mackerel Pony fish Yellowtail scad Yellowstripe scad Black pomfret Dory

According to Al-Sebeih et al., a lateral neck X-ray has 25% sensitivity and 86.3% specificity for the detection of the impacted fish bone (31). If there is a suspicion of ingested FB, esophagoscopy or laryngoscopy examinations are usually regarded as the methods of choice, because they can also be used as therapeutic tools (52). Mucosal changes like edema, laceration, or ulceration may indicate the migration of the FB outside the esophagus. In cases involving persistent neck pain and a history of FB ingestion, if the results of the lateral X-ray and endoscopy are negative, the CT scan should be performed. The study by Goh et al. shows the superiority of CT over clinical history and plain X-ray (18). According to Cavelier et al. CT has a sensitivity of 100% in detecting FBs in soft tissues (42). Moreover, it is very useful for evaluating complications. Shrime et al. claim that for a patient with suspicion of migrated FB, initial CT scanning is the most cost-effective diagnostic strategy (69). In addition, CT can provide important information to guide the surgical approach (35) (**Figure 5**).

**Figure 5:**
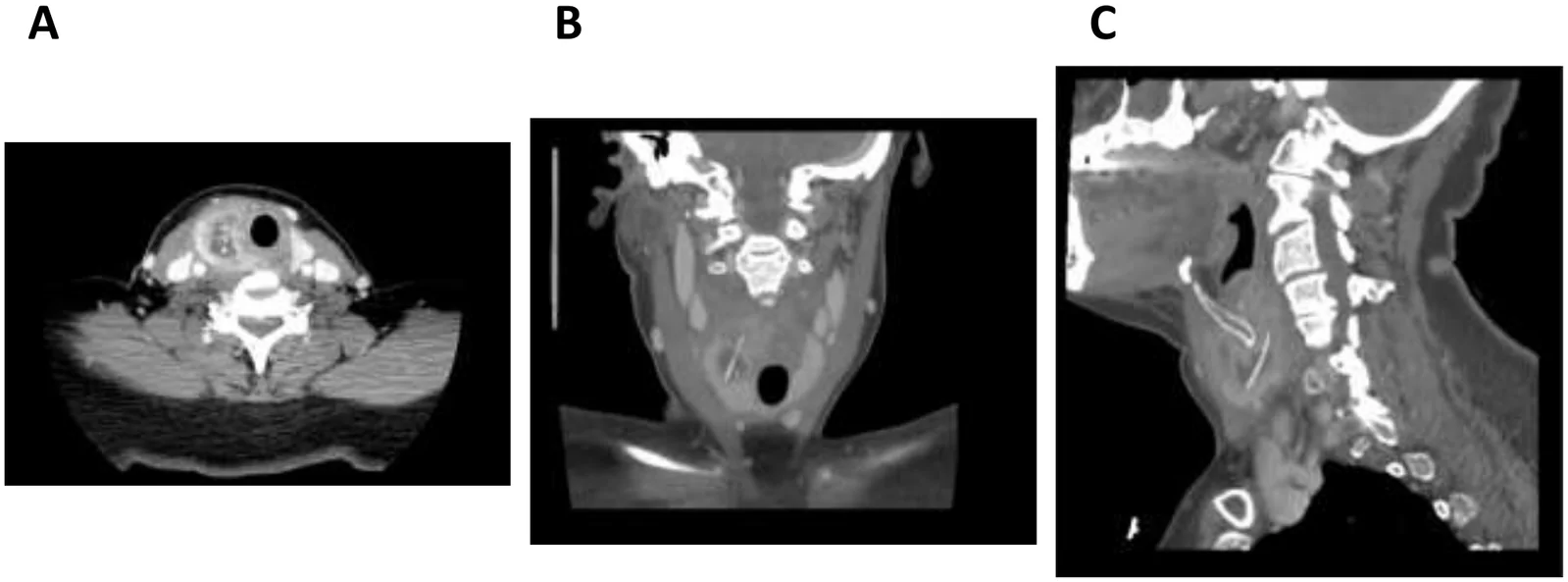
Computed tomography images of a migrated fish bone embedded in the thyroid gland. **(A)** Axial plane. **(B)** Coronal plane. **(C)** Sagittal plane. Panels **(A–C)** were adapted from AlZarouni N, et al. (49). Licensed under CC BY 4.0 (https://creativecommons.org/licenses/by/4.0/).

Kotecha et al. point out that most solid FBs show acoustic shadows, so cervical US might be as good as a CT scan in detecting FB in the thyroid gland (7) (**Figure 6**). Petrarolha et al. used US as the first-line method in the detection of the FB of the thyroid gland (40). Watanabe et al. have used the US intraoperatively during surgery to carefully examine the gland (34).

**Figure 6:**
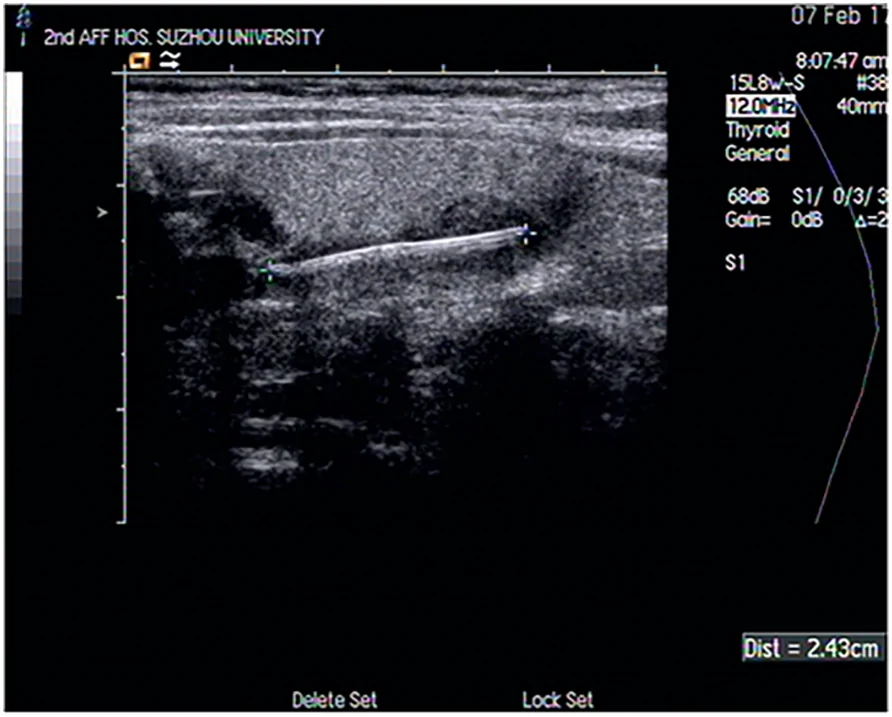
Ultrasound appearance of a migrated fish bone embedded in the thyroid gland from Wu et al. (41). Reproduced from *Migratory Fish Bone in the Thyroid Gland: Case Report and Literature Review* (*Case Reports in Medicine*), by E. Wu, L. Huang, Y. Zhou, and X. Zhu, 2018, https://doi.org/10.1155/2018/7345723. Used under Creative Commons Attribution (CC-BY) license.

Despite the fact that in most of the reviewed patients no complications were observed, abscess formation was the most common and important problem. Interestingly, one case of thyroid gland-cutaneous fistula secondary to the migratory FB was reported (35). This patient described by Ohbuchi et al. had a 2-month history of a painless cutaneous pus discharge in her anterior neck.

A case of hyperthyroidism resembling subacute thyroiditis following the penetration of the thyroid gland by a fish bone was described by Gokon et al. (48). A 51-year-old patient was admitted to the hospital due to neck pain, an FB sensation in her throat, and hyperthyroidism in the laboratory tests with suspected subacute thyroiditis. The treatment with prednisolone was initiated. However, after 4 days of persisting fever, a CT of the neck was performed, showing a fish bone in the left thyroid lobe. Conservative treatment with antibiotics was started, resulting in an improvement of the symptoms. Subsequent CT and US detected no fish bone, so the surgery was canceled. During follow-up observation temporary hypothyroidism was observed. Finally, after 7 weeks the laboratory tests returned to reference ranges.

The dominant treatment method for the FBs in the thyroid gland is indisputably surgical removal. When the abscess is formed, drainage is recommended (51). The decision about thyroid-preserving surgical removal or hemithyroidectomy should be made individually by the surgeon based on the exact location, complications, and general state of the patient. We have found only one case of total thyroidectomy due to the FB migration, but the decision to extend the procedure was made due to the incidental finding of the large goiter (31). It should be noted that minimally invasive endoscopic methods have also been described in the literature and can be applied in selected cases (33). Li et al. emphasized that after removing an FB endoscopically, it is necessary to perform a follow-up gastroscopy to check for complications such as bleeding or perforation (6).

Sometimes FB in the thyroid gland might be iatrogenic. Teflonoma is a term regarding the formation of a granulomatous FB reaction in a tissue to Teflon particles (17). Lavon et al. described a case of teflonoma formation in the thyroid gland of the patient, who underwent teflon endoscopic injection in the treatment of unilateral vocal cord paralysis (29). A similar situation with an FB reaction in the thyroid gland, but caused by ultrasound gel, was reported by Adeoti et al. (1). In this case, the patient presented with neck pain and swelling of the thyroid gland after undergoing a fine-needle aspiration biopsy (FNAB). The authors claim that they were uncertain what FB could be introduced into the gland, but the ultrasound gel was the most suspicious. Another iatrogenic cause of abscess formation in the thyroid gland was the minitracheostomy tube and puncture needle (8, 19). Orobello et al. revealed an unusual case of migration of a piece of vegetable into the thyroid gland through a congenital fourth branchial cleft fistula (43). The formation of the abscess occurred, so the hemithyroidectomy was performed. In this situation, an FB migrated to the thyroid gland through the congenital tract by itself. It was one of only two published cases of patients under the age of eighteen. The second one concerned a 12-year-old boy, who was working in a factory (20). During cutting iron rods with a chisel and hammer, a metallic splinter penetrated through his neck. A surgical intervention was needed, luckily without the need for hemithyroidectomy.

The majority of the publications concerning FBs in the thyroid gland cite the article by Jemerin and Aronoff as the first one to mention this problem in the scientific literature (9). However, we found the short report written by Wolfson in 1926, in which the author described a case of a woman who was treated with repeated injections of a solution of quinine urea hydrobromide due to a growing goiter (8). After about two years of treatment, when the consistency of the gland was hard because of progressive fibrosis, the patient suddenly shook her head during the procedure, causing the needle to break deep in her gland. After three months of observation, she developed symptoms of acute thyroiditis, and the subtotal thyroidectomy had to be performed.

### 4.2 Foreign body reactions in post-thyroidectomy bed

Noteworthy, especially for the clinicians performing neck US, sometimes in the bed after thyroidectomy, lesions mimicking thyroid regrowth may be visible. A summary of the US findings and FNAB results of the FB reactions in the post-thyroidectomy bed is shown in **Table 3**.

**Table 3:** Foreign body reactions in the post-thyroidectomy bed (70–82).

No.	Material	US findings	FNAB findings
1.	Oxidised cellulose	- hypo-or isoechogenic lesions - well-defined margins - no vascularisation	- small cellular structures consisting of oxidised cellulose - inflammatory components with macrophages in some cases - degenerated debris
2.	Absorbable gelatin	- uniform elongated isoechogenic lesions - thin hypoechoic halo - no vascularisation	- multinucleated giant cells - macrophages
3.	Silk suture	- heterogeneous nodule with hypoechogenic rim - irregular shape - hypoechogenic collections containing hyperechogenic lines - knot and ear sign	- neutrophils, histiocytes, granulation tissue - foreign body type giant cells
4.	Polytetrafluoroethylene	N/A	N/A
5.	Port-site pressure injury caused by endoscopic surgical instruments	- isoechogenic homogenous nodule - calcifications with distal shadowing	- necrotic material - foamy macrophages - multinucleated giant cells

N/A, not available.

Sebastian-Valles et al., in their publication, described 15 cases of the development of FB reactions to oxidised cellulose (OC) (70). OC is a local haemostatic biomaterial widely used in thyroid cancer surgery. The lesions visible during the follow-up US examinations were hypo- or isoechogenic with clear borders and no vascularisation. The authors analyzed FNAB results of the structures visualized in the thyroid bed. The results showed small cellular forms consisting of OC and macrophages. Another illustration of FB reaction to OC was described in the article by Shahin et al. (71). Interestingly, in the similar case described by Liberale et al., the diagnostic modalities, including US, CT, and FNAB, could not exclude the recurrence of papillary thyroid cancer, so surgery was needed (72). Royds et al. described the case of a patient after thyroidectomy with wound swelling, erythema, and discharge from the surgical site, where OC reaction mimicked an abscess (73). Alameer highlights that it is important to document the use of OC during the procedure, and it is recommended to use the minimal amount necessary to reach hemostasis (74). Another haemostatic material capable of causing an FB reaction in the post-thyroidectomy bed can be an absorbable gelatin compressed sponge. Tublin et al. report that sonographic characteristics of such “lesions” include elongated shape, isoechogenity, thin hypoechoic halo, no calcifications, and no vascularisation (75). According to the authors, those changes are better visible shortly after surgery and probably resolve over time. Hernández-Bonilla et al. underscore that FNAB should be performed in the suspicious cases (76).

In the article by Soylu et al., two cases of silk suture reaction after thyroidectomies with the formation of granuloma were reported (77). The US showed heterogeneous, indeterminate nodules in the beds after thyroidectomies. Cytologic evaluations manifested neutrophils, histiocytes, and granulation tissue. There are a few more similar case reports in the literature (78, 79). Hocwald et al. pointed out that intradermal skin tests might be a good predictor of allergy to sutures, which is probably the underlying cause of the FB reaction (83). Interestingly, Eldridge et al., as early as 1987, conducted a retrospective survey in which they analyzed the incidence of granulomas in the bed after thyroid surgeries (84). It occurred that this pathology has happened in 8% of cases when silk sutures were used during the procedures. On the contrary, no cases of granulomas were reported when the absorbable sutures were applied. In conclusion to their article, the authors stated that due to significant morbidity, the use of silk in the neck should be abandoned. However, Ibrahim et al. reported a case of an FB reaction to the absorbable sutures in the bed after thyroidectomy (85). Suh et al. in their publication compared the sonographic findings of suture granulomas with thyroid cancer recurrence after thyroidectomy (80). The first mentioned group had a more frequent irregular shape, markedly hypoechoic echogenity, a heterogenous echotexture, and no vascularity. Furthermore, the potential pathognomic finding of suture granuloma was the presence of internal echogenic foci resembling the silhouette of Mickey Mouse; however, the authors termed this finding the “knot-and-ear sign.” This finding was found in 50% of suture granuloma cases, whereas it was not observed in recurrent tumors at all.

Jung et al. presented two patients with increased uptake of FDG in PET/CT imaging during postthyroidectomy follow-up (81). The surgery was performed endoscopically via the axillo–breast approach. FNAB of the suspected structures was performed. The histopathological examination showed granulomas caused by an FB reaction, supposedly by the pressure and irritation of the chest wall tissues caused by surgical instruments. Yeretsian et al. described a similar case of false-positive PET interpretation in the bed after thyroidectomy (82). This time it was caused by a teflonoma, which occurred after a teflon injection into the paralyzed vocal cords due to the left laryngeal recurrent nerve paralysis after surgery.

### 4.3 Limitations

This review has several limitations. The chief limitation is the fact that the available evidence is limited by the extreme rarity of FBs in the thyroid gland. The only sources are case reports and small case series, which represent a low level of evidence and limit the ability to draw definitive conclusions. Additionally, some case reports do not contain complete data. Consequently, the findings are subject to publication bias. Furthermore, heterogenity in reporting and management strategies limits direct comparisons between cases.

## 5 Conclusions

FBs in the thyroid gland and FB reactions in the post-thyroidectomy bed represent rare challenges that require prompt management. In principle, all FBs in the thyroid gland should be treated as emergencies. The consequences of migratory FB may be potentially fatal. Physicians should be aware that FBs can be stuck in the thyroid gland, especially if the patient presents with unilateral neck pain and/or swelling at the initial evaluation. Endocrine dysfunction might be seen very rarely. To prevent misdiagnosis, a detailed medical history, followed by physical examination and different diagnostic methods, is vital. CT scanning is the most sensitive tool to detect FBs in the thyroid gland. Additionally, it is very useful for evaluating complications and surgical planning. Surgical removal with or without hemithyroidectomy remains the main treatment method. In some selected cases, minimally invasive endoscopic techniques might be possible. It should be emphasized that in patients after thyroidectomy, the differentiation between FB reaction and local tumor recurrence requires cautious interpretation of the results of diagnostic tests. FNAB should be performed in the suspicious cases. Clinicians need to be aware of the appearance and symptoms of such FB reactions to avoid misdiagnosis. Due to the limitations of this review, the conclusions should be interpreted with caution. Further analysis of well-documented cases is needed to better characterize the clinical presentation and optimal management of the patients with this rare condition.
