# Supplementary material for: Foreign bodies in the thyroid gland and foreign body reactions in the post-thyroidectomy bed
Source: Front Endocrinol (Lausanne). 2026 Sep 16;17:1826008. doi: 10.3389/fendo.2026.1826008 (PMC13624971; doi:10.3389/fendo.2026.1826008)
Supplement: Supplementary file 1 [file Table1.docx]

Supplementary Material

Supplementary Table S1. Data of sixty-three previously reported cases of foreign bodies in the thyroid gland.

| No. | First author | Year of publication | Foreign body | Size [mm] | Sex | Age | Country | Chief complaints | Time since first symptoms to hospital presentation | Misdiagnosis at the initial evaluation? | Location | Main Complications | Main treatment method | Endoscopy | Diagnostic methods | Reference |
| --- | --- | --- | --- | --- | --- | --- | --- | --- | --- | --- | --- | --- | --- | --- | --- | --- |
| 1 | Wolfson | 1926 | Puncture Needle | 25 | F | 58 | USA | Dyspnea, neck swelling | 3 months | No | Right lobe | Abscess | Subtotal thyroidectomy | No | None | 8 |
| 2 | Jemerin | 1949 | Chicken bone | N/A | F | 58 | USA | N/A | 7 days | N/A | Right lobe | Abscess | Hemithyroidectomy | N/A | N/A | 9 |
| 3 | Jemerin | 1949 | Chicken bone | N/A | F | 73 | USA | N/A | 8 days | N/A | Right lobe | Abscess | Hemithyroidectomy | N/A | N/A | 9 |
| 4 | Jemerin | 1949 | Fish bone | N/A | F | 61 | USA | N/A | 21 days | N/A | Left lobe | Abscess | Hemithyroidectomy | N/A | N/A | 9 |
| 5 | Sanfilippo | 1980 | Polytetrafluoroethylene | 20 | F | 65 | USA | Neck swelling | 17 months | No | Right lobe | None | Thyroid-preserving surgical removal | No | None | 10 |
| 6 | Wilson | 1987 | Polytetrafluoroethylene | 22 | F | 31 | USA | Neck swelling | 3 months | No | Left lobe | None | Hemithyroidectomy | No | None | 11 |
| 7 | al Muhanna | 1990 | Fish bone | 35 | F | 42 | Kuwait | Odynophagia, neck pain | Few hours | No | Left lobe | Abscess | Hemithyroidectomy | Yes | X-ray, CT | 12 |
| 8 | Bendet | 1992 | Fish bone | 26 | F | 39 | Israel | Dysphagia, neck pain | 5 days | Yes | Left lobe | N/A | Thyroid-preserving surgical removal | Yes | Barium fluoroscopy, X-ray, US, CT | 13 |
| 9 | Foo | 1993 | Fish bone | N/A | F | 72 | Singapore | Throat discomfort, neck swelling | 7 days | No | Left lobe | Abscess | Thyroid-preserving surgical removal | Yes | X-ray, CT | 14 |
| 10 | Foo | 1993 | Fish bone | N/A | F | 66 | Singapore | Throat discomfort, Neck swelling, neck pain | 7 weeks | Yes | Left lobe | None | Thyroid-preserving surgical removal | Yes | X-ray, CT | 14 |
| 11 | Coret | 1993 | Fish bone | N/A | F | 38 | Israel | Dysphagia, odynophagia, Neck swelling | 5 days | Yes | Left lobe | Abscess | Thyroid-preserving surgical removal | Yes | Barium fluoroscopy, X-ray, US | 15 |
| 12 | Chowdhury | 1993 | Metal needle | N/A | M | 37 | United Kingdom | Throat discomfort, neck pain | Few years | No | Left lobe | None | Thyroid-preserving surgical removal | Yes | Barium fluoroscopy, X-ray, CT | 16 |
| 13 | Wassef | 1994 | Polytetrafluoroethylene | 18 | F | 67 | France | Neck swelling | 3 years | No | Isthmus | None | Thyroid-preserving surgical removal | No | US, CT | 17 |
| 14 | Goh | 1999 | Fish bone | 30 | F | 68 | Singapore | None | 4 days | N/A | Right lobe | None | Thyroid-preserving surgical removal | Yes | X-ray, CT | 18 |
| 15 | Goh | 1999 | Fish bone | 35 | F | 65 | Singapore | Neck swelling | 14 days | N/A | Left lobe | None | Thyroid-preserving surgical removal | No | X-ray, CT | 18 |
| 16 | Goh | 1999 | Fish bone | 25 | F | 66 | Singapore | Throat discomfort, neck pain, neck swelling | 7 weeks | N/A | Left lobe | Abscess | Thyroid-preserving surgical removal | Yes | X-ray, CT | 18 |
| 17 | Goh | 1999 | Fish bone | 25 | F | 72 | Singapore | Throat discomfort, neck swelling | 7 days | N/A | Left lobe | Abscess | Thyroid-preserving surgical removal | Yes | X-ray, CT | 18 |
| 18 | Browne | 1999 | Minitracheostomy tube | N/A | F | 72 | Ireland | Neck swelling | 1 day | No | N/A | Abscess | Manually removed | No | None | 19 |
| 19 | Chakraborty | 1999 | Metal wire | 17 | M | 12 | India | Neck pain | N/A | No | Right lobe | None | Thyroid-preserving surgical removal | No | US, X-ray | 20 |
| 20 | Arumainathan | 2000 | Fish bone | 20 | F | 38 | Malaysia | Dysphagia | 5 days | No | Left lobe | None | Thyroid-preserving surgical removal | Yes | X-ray, CT | 21 |
| 21 | Sergi | 2002 | Fish bone | 25 | F | 59 | Italy | Neck pain, Foreign body sensation | N/A | No | Left lobe | None | Thyroid-preserving surgical removal | Yes | US, X-ray, CT | 22 |
| 22 | Kumar | 2003 | Metal needle | 30 | F | 35 | India | Neck pain, Foreign body sensation | 3 days | No | Right lobe | None | Thyroid-preserving surgical removal | Yes | X-ray, CT | 23 |
| 23 | Sreetharan | 2004 | Tooth | N/A | M | 44 | Malaysia | Dysphagia, fever | 7 days | No | Right lobe | None | Thyroid-preserving surgical removal | Yes | CT | 24 |
| 24 | Masuda | 2005 | Fish bone | 27 | F | 61 | Japan | Dysphagia, Neck pain | 16 days | Yes | Left Lobe | none | Thyroid-preserving surgical removal | Yes | X-ray, CT, US | 25 |
| 25 | Astl | 2005 | Metal wire | 25 | F | 21 | Czech Republic | N/A | N/A | N/A | Right lobe | N/A | Thyroid-preserving surgical removal | N/A | X-ray,CT | 26 |
| 26 | Zohra | 2006 | Metal wire | N/A | F | 26 | Pakistan | Neck pain | 3 weeks | No | Right lobe | None | Thyroid-preserving surgical removal | Yes | X-ray, CT | 27 |
| 27 | Lin | 2008 | Fish bone | 30 | M | 71 | China | Foreign body sensation, Neck pain, Fever | 10 days | Yes | Right lobe | Abscess | Hemithyroidectomy | Yes | Barium fluoroscopy, US | 28 |
| 28 | Lavon | 2008 | Polytetrafluoroethylene | 30 | F | 55 | Israel | Dyspnea, hoarseness | 4 years | No | Left Lobe | None | Observation | Yes | CT | 29 |
| 29 | Hohman | 2010 | Metal wire | 30 | F | 34 | USA | Neck pain, odynophagia | 3 days | Yes | Right lobe | Abscess | Endoscopic removal | Yes | CT | 30 |
| 30 | Al.-Sebeih | 2010 | Fish bone | N/A | F | 28 | Kuwait | Neck pain, fever, neck swelling | 3 days | No | Right lobe | Abscess | Hemithyroidectomy | Yes | X-ray, CT | 31 |
| 31 | Al.-Sebeih | 2010 | Fish bone | N/A | F | 56 | Kuwait | Neck pain | 2 months | Yes | Left lobe | None | Thyroidectomy | Yes | X-ray, CT | 31 |
| 32 | Chen | 2011 | Fish bone | 41 | F | 50 | Taiwan | Neck pain, neck swelling | 1 month | Yes | Right lobe | Abscess | Thyroid-preserving surgical removal | Yes | X-ray, CT | 32 |
| 33 | DePietro | 2012 | Fish bone | 20 | M | 58 | USA | Neck pain | 7 day | Yes | Right lobe | Abscess | Endoscopic removal | yes | X-ray, CT | 33 |
| 34 | Watanabe | 2012 | Fish bone | 34 | F | 69 | Japan | Neck swelling | 9 months | No | Right lobe | None | Thyroid-preserving surgical removal | No | US, CT | 34 |
| 35 | Ohbuchi | 2012 | Fish bone | 28 | F | 56 | Japan | Pus discharge | 1 year | Yes | Left lobe | Cutaneous fistula in the anterior neck | Hemithyroidectomy | Yes | CT | 35 |
| 36 | Chen | 2013 | Metal wire | 28 | F | 38 | China | Odynophagia | Few hours | No | Right lobe | None | Thyroid-preserving surgical removal | Yes | CT | 36 |
| 37 | Kotecha | 2014 | Fish bone | 40 | M | 35 | India | Dysphagia | 15 days | No | Left lobe | None | Thyroid-preserving surgical removal | Yes | X-ray, CT, US | 7 |
| 38 | Shivappa | 2015 | Metal wire | 6 | M | 36 | India | N/A | Few hours | No | Right lobe | None | Thyroid-preserving surgical removal | No | CT | 37 |
| 39 | Johari | 2016 | Fish bone | 40 | F | 80 | Malaysia | Neck pain, odynophagia, dysphagia | 6 days | No | Right lobe | Abscess | Thyroid-preserving surgical removal | Yes | X-ray, CT | 38 |
| 40 | Dwivedi | 2016 | Lamb bone | 40 | M | 40 | United Kingdom | Neck pain, odynophagia | Few hours | Yes | Left lobe | Abscess | Thyroid-preserving surgical removal | Yes | X-ray | 39 |
| 41 | Petrarolha | 2017 | Fish bone | N/A | F | 67 | Brasil | Neck pain, Dysphagia | 9 days | No | Left Lobe | Abscess | Thyroid-preserving surgical removal | No | US, CT | 40 |
| 42 | Wu | 2018 | Fish bone | 25 | F | 31 | China | Foreign body sensation, mild tenderness | 2 months | Yes | Left lobe | None | Hemithyroidectomy | Yes | US | 41 |
| 43 | Cavelier | 2019 | Fish bone | 28 | F | 59 | Belgium | Neck pain, odynophagia, sialorrhea, neck swelling | 3 days | Yes | Left lobe | None | Hemithyroidectomy | Yes | US, CT | 42 |
| 44 | Orobello | 2020 | Vegetable matter | N/A | F | 17 | USA | Throat discomfort, dysphagia | 4 weeks | No | Right lobe | Abscess | Hemithyroidectomy | Yes | US, CT | 43 |
| 45 | Wang | 2020 | Metal needle | 20 | F | 42 | China | Neck pain | 1 day | No | Right lobe | None | Thyroid-preserving surgical removal | Yes | CT, X-ray | 44 |
| 46 | Fakoury | 2020 | Fish bone | 25 | F | 49 | UAE | Neck pain, dysphagia | 15 days | No | Right lobe | Abscess | Thyroid-preserving surgical removal | Yes | X-ray, US, CT | 45 |
| 47 | Huang | 2022 | Fish bone | 23 | F | 60 | Taiwan | Foreign body sensation, odynophagia | 10 days | Yes | Left Lobe | Hematoma | Hemithyroidectomy | Yes | CT | 46 |
| 48 | Hendricks | 2022 | Fish bone | 26 | F | 71 | Germany | Foreign body sensation, neck pain, fever, odynophagia | 4 days | Yes | Right lobe | Purulent thyroiditis, sepsis | Hemithyroidectomy | Yes | US, CT | 47 |
| 49 | Gokon | 2022 | Fish bone | 25 | F | 51 | Japan | Neck pain, foreign body sensation, subfebrile state | 1 day | Yes | Left lobe | Hyperthyroidism | Conservative non-surgical treatment | Yes | US, CT | 48 |
| 50 | AlZarouni | 2022 | Fish bone | N/A | F | 49 | Egypt | Neck pain, neck swelling, dysphagia | 2 weeks | No | Right lobe | Abscess | Thyroid-preserving surgical removal | Yes | CT | 49 |
| 51 | Adeoti | 2023 | Ultrasound gel | N/A | F | 33 | USA | Neck pain, neck swelling, odynophagia | 3 weeks | No | Right lobe | Thyroiditis | Hemithyroidectomy | Yes | US, CT | 1 |
| 52 | Aparicio-Lopez | 2023 | Chicken bone | N/A | M | 42 | Spain | Foreign body sensation, dysphagia, fever, neck swelling | 3 days | No | Left lobe | N/A | Hemithyroidectomy | No | CT | 50 |
| 53 | Foul | 2023 | Fish bone | 35 | F | 78 | United Arab Emirates | Throat pain, odynophagia | Few hours | Yes | Left lobe | None | Thyroid-preserving surgical removal | Yes | X-ray, CT | 51 |
| 54 | Sun | 2023 | Fish bone | 25 | F | 52 | China | Throat pain | 8 days | Yes | Left lobe | None | Thyroid-preserving surgical removal | Yes | CT, US | 52 |
| 55 | Abdul Rashid | 2023 | Fish bone | 20 | F | 52 | Malaysia | Neck pain | 4 days | No | Right lobe | None | Thyroid-preserving surgical removal | Yes | CT | 53 |
| 56 | Soribe | 2023 | Fish bone | 40 | F | 76 | Poland | Odynophagia | 3 days | No | Left lobe | Abscess | Endoscopic removal | Yes | CT | 54 |
| 57 | Yang | 2024 | Fish bone | 35 | M | 57 | China | Throat pain, dysphagia | N/A | No | Left Lobe | None | Endoscopic removal | Yes | CT | 55 |
| 58 | AlTarayra | 2024 | Fish bone | N/A | F | 60 | Palestine | Odynophagia | 10 days | No | Left lobe | None | Hemithyroidctomy | No | X-ray, CT | 56 |
| 59 | Kong | 2024 | Piece of glass | 20 | F | 71 | New Zealand | Throat pain, dysphagia, odynophagia | 4 days | No | Left lobe | Abscess | Hemithyroidectomy | Yes | X-ray,CT | 57 |
| 60 | Li | 2024 | Fish bone | 30 | F | 70 | China | Foreign body sensation | 20 days | No | Left lobe | None | Thyroid-preserving surgical removal | Yes | CT | 58 |
| 61 | Ouédraogo | 2024 | Fish bone | 20 | F | 37 | India | Neck swelling | 8 months | No | Left lobe | None | Thyroid-preserving surgical removal | No | US | 59 |
| 62 | Kuang | 2025 | Fish bone | 30 | F | 38 | China | Neck pain, Foreign body sensation | Few hours | Yes | Right lobe | None | Thyroid-preserving surgical removal | Yes | CT, US | 60 |
| 63 | Lee | 2025 | Fish bone | 25 | F | 60 | South Korea | Neck pain, odynophagia | 1 hour | No | Right lobe | None | Thyroid-preserving surgical removal | Yes | CT | 61 |

N/A – not available, F- female, M-male, X-ray- plain X-ray, CT- computed tomogarphy, US- ultrasound examination.
